# Aldo-keto reductases: Role in cancer development and theranostics

**DOI:** 10.32604/or.2024.049918

**Published:** 2024-07-17

**Authors:** SIDDAVARAM NAGINI, PRATHAP REDDY KALLAMADI, KRANTHI KIRAN KISHORE TANAGALA, GEEREDDY BHANUPRAKASH REDDY

**Affiliations:** 1 World Neem Organization, Mumbai, 400101, India; 2 Department of Biochemistry, ICMR-National Institute of Nutrition, Hyderabad, 500007, India; 3 Department of Pathology and Cell Biology, Columbia University Irving Medical Center, New York, 10032, USA

**Keywords:** Aldo-keto reductases (AKRs), Aldo-keto reductase (AKR) inhibitors, Cancer, Drug-resistance, Xenobiotics

## Abstract

Aldo-keto reductases (AKRs) are a superfamily of enzymes that play crucial roles in various cellular processes, including the metabolism of xenobiotics, steroids, and carbohydrates. A growing body of evidence has unveiled the involvement of AKRs in the development and progression of various cancers. AKRs are aberrantly expressed in a wide range of malignant tumors. Dysregulated expression of AKRs enables the acquisition of hallmark traits of cancer by activating oncogenic signaling pathways and contributing to chemoresistance. AKRs have emerged as promising oncotherapeutic targets given their pivotal role in cancer development and progression. Inhibition of aldose reductase (AR), either alone or in combination with chemotherapeutic drugs, has evolved as a pragmatic therapeutic option for cancer. Several classes of synthetic aldo-keto reductase (AKR) inhibitors have been developed as potential anticancer agents, some of which have shown promise in clinical trials. Many AKR inhibitors from natural sources also exhibit anticancer effects. Small molecule inhibitors targeting specific AKR isoforms have shown promise in preclinical studies. These inhibitors disrupt the activation of oncogenic signaling by modulating transcription factors and kinases and sensitizing cancer cells to chemotherapy. In this review, we discuss the physiological functions of human AKRs, the aberrant expression of AKRs in malignancies, the involvement of AKRs in the acquisition of cancer hallmarks, and the role of AKRs in oncogenic signaling, and drug resistance. Finally, the potential of aldose reductase inhibitors (ARIs) as anticancer drugs is summarized.

## Introduction

Cancer, a multifactorial heterogeneous group of diseases, is the second leading cause of mortality worldwide [1]. The process of neoplastic transformation involves the progressive accumulation of genetic and epigenetic changes in vital genes that regulate cell survival, proliferation, differentiation, apoptosis, and genome integrity. Dysregulated cellular signaling plays a key role in enabling the acquisition of hallmark traits of cancer [2,3]. Despite the development of state-of-the-art technologies in diagnosis and novel therapeutic modalities, morbidity, and mortality due to cancer continue to be a major challenge to oncologists due to the high rate of recurrence and chemoresistance [4,5]. Several mechanisms contribute to therapy resistance such as xenobiotic-metabolism, drug efflux, and apoptosis evasion, among several others [6].

Both cytochrome P450 (CYP) and non-CYP pathways are involved in phase I xenobiotic metabolism. Although a vast majority of xenobiotics are primarily metabolized by the CYP enzymes, flavin monooxygenase, esterases, and aldo-keto reductases (AKRs) are also involved in the detoxication of drugs and xenobiotics. The CYP monooxygenases are membrane-bound hemeproteins that act on a wide variety of xenobiotics, whereas the AKRs are cytosolic oxidoreductases that primarily catalyze redox transformations of carbonyl-containing xenobiotics [7]. CYPs as well as AKRs that play a critical role in regulating the efficacy of drugs are also implicated in chemoresistance [8,9]. Inhibitors of CYPs and AKRs have therefore emerged as attractive candidate agents for cancer therapy and to counter chemoresistance [10].

The AKR protein superfamily of nicotinamide-adenine dinucleotide phosphate (NADP)-dependent enzymes play a central role in the reduction of a variety of aldehydes and carbonyls formed during drug metabolism and detoxification of xenobiotics. The AKR superfamily consists of over 190 members grouped into 16 distinct families, AKR1 to AKR16 based on amino acid sequence identity, with each family comprised of many subfamilies. In humans, 15 different types of AKRs have been identified that belong to three main families, AKR1, AKR6, and AKR7. Of these, the AKR1 family is the most well-characterized and is comprised of three members, AKR1B1, AKR1B10, and AKR1B15, which share over 70% sequence identity and substrate specificity [11].

The nomenclature of AKR proteins begins with the root symbol of AKR, followed by an Arabic number that indicates the family, a letter indicating the subfamily, and another Arabic number for the specific protein. For instance, in AKR1B1, the first Arabic number (AKR1) represents the family, the letter following the first number (AKR1B) indicates the subfamily, while the second Arabic number (AKR1B1) represents the individual member protein [12].

The AKRs are ubiquitously distributed in both prokaryotes and eukaryotes. AKR1B1 also known as aldose reductase (AR, EC 1.1.1.21) is expressed in many tissues, with high expression in the adrenal gland, seminal vesicles, placenta, eye lens, retina, kidney, and cells of the gastrointestinal tract. AKR1B1 expression was found to be low in the liver, prostate, testes, and thymus, and absent in most normal human tissues [13,14]. High expression of AKR1B10, also known as aldose reductase-like-1 (ARL-1), is seen in the adrenal glands, small intestine, and colon [11,15]. The expression of AKR1B15, a novel enzyme that shares 92% amino acid sequence homology with AKR1B10, is generally low in most tissues and limited to the adipose tissue, testis, and placenta [16].

AKR1B1 (AR), the most extensively studied AKR, catalyzes the nicotinamide adenine dinucleotide phosphate reduced (NADPH)-dependent reduction of aldehydes and ketones to their corresponding alcohols. The reduction of glucose to sorbitol followed by conversion to fructose is depicted as the polyol pathway. The reduction of glucose to fructose by AR was first identified in the seminal vesicle by Hers in 1956 [17]. Subsequently, van Heyningen [18] demonstrated the accumulation of galactitol and sorbitol in the rat lens during diabetic cataractogenesis. Accumulation of polyols in the lens was believed to enhance intracellular osmotic pressure leading to over-hydration, and imbalance in Na^+^/K^+^ homeostasis [19]. Based on these observations, AR was implicated as the main facilitator of hyperglycemic injury. A growing body of evidence recognized AR as a key mediator of secondary diabetic complications such as retinopathy, cataracts, microangiopathy, neuropathy, and nephropathy [20,21].

AKRs are overexpressed in several inflammation-associated pathological conditions such as alcoholic liver cirrhosis, atherosclerosis, asthma, gout, osteoporosis, uveitis, sepsis, and cancer [22–24]. While AKR1B1 is implicated in diabetic complications, AKR1C1-1C3 are involved in steroid hormone-dependent tumors, AKR1D1 in bile acid deficiency, and AKR1B10 in defects in retinoic acid signaling [11,15,25,26]. Overexpression of AKRs has been extensively reported in several malignant tumors [15,27–31].

Several studies support the tenet that inhibition of AR ameliorates inflammatory diseases besides diabetic complications [22,32,33]. Consequently, several synthetic AR inhibitors (ARIs) such as sorbinil, zopolrestat, fidarestat, and epalrestat have been tested for their efficacy in addressing these conditions [33,34]. While numerous synthetic ARIs have demonstrated excellent inhibition and shown promising results in animal studies, their efficacy in clinical trials was not very encouraging with some ARIs exhibiting adverse side effects. Hence, of late, natural products are being extensively investigated for their ability to inhibit AR to overcome toxicity and adverse side effects of synthetic agents [34,35].

In this review, we discuss the physiological functions of human AKRs, the aberrant expression of AKRs in malignancies, the involvement of AKRs in the acquisition of cancer hallmarks, and the role of AKRs in oncogenic signaling, and drug resistance. Finally, the potential of ARIs as anticancer drugs is summarized.

## Structure

The AKR1B1 gene (18 kb) localized on chromosome 7q33 contains 10 exons and 9 introns. The promoter region consists of a TATA (TATTTA) box at −37 and a CCAAT box at −104. Two Alu repeats are present in intron 1, 4, and 9, respectively. Additionally, an androgen-like response element (ALRE), three osmotic response elements (ORE), an activator protein 1 (Ap-1) binding site, and a thyroid receptor element (TRE) are located upstream of the gene. The AKR1B1 gene is translated to a monomeric protein containing 316 amino acid residues [26,36]. Fig. 1A provides a schematic illustration of the structure of the AKR1B1 gene.

**Figure 1A:**
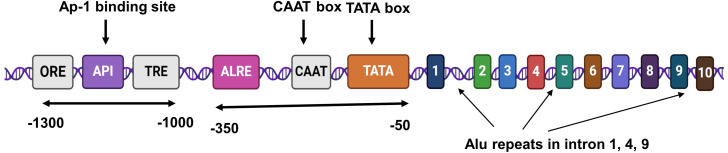
A schematic representation of AKR1B1 gene structure.

The AKR1B10 gene (13.8 kb) contains 10 exons and 9 introns and shares close structural similarities with the AKR1B1 gene [37]. However, unlike the AKR1B1 gene, the AKR1B10 gene promoter contains a TATA-like (TAATAA) box from −145 to −140 bp and ORE is absent. The CAAT box is present in the region spanning from −193 to −190 bp. Multiple protein binding sites including those for p53, c-Ets-1, C/EBP, and AP-1 have been identified in the AKR1B10 promoter. Luciferase reporter analyses revealed that the sequences between −530 and −520 bp contain functional ARE sequences in tandem with the AP-1 site [38].

The AKRs are cytosolic monomeric proteins of molecular weight 30–40 kDa. Almost all AKRs share a common protein structure, comprised of an (α/β)8-barrel with alternating eight α-helices and eight β-strands and a pyridine nucleotide binding site [39]. The α/β barrel motif is essential for oligomerization as well as for binding of cofactors and metals, thereby generating an active site geometry (Fig. 1B). The back of the barrel consists of three large loops that are responsible for substrate specificity [25]. The binding site of NADPH is highly conserved and involves the hydrophobic amino acids Tyr-48, His-110, Cys-298, Trp-20, and Phe-122 in the active site of the enzyme [40–42]. Both AKR1B1 and AKR1B10 possess a negatively charged acidic hydroxyl group (Tyr48) in the catalytic site that may be important in structure-based drug design [43].

**Figure 1B:**
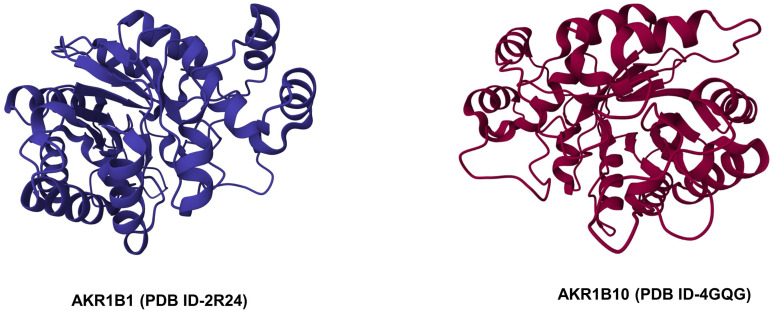
Crystal structures of AKR1B1 and AKR1B10 (Source: RCSB PDB).

## Physiological Functions of Human AKRs

AKRs are pluripotent enzymes that act on diverse substrates and catalyze redox transformations during intermediary metabolism and detoxification [11,44]. AKRs primarily function as reductases by utilizing the reducing equivalents from NADPH. AKRs are involved in the metabolism of sugar aldehydes, lipid aldehydes, keto-prostaglandins, retinals, quinones, keto-steroids, carcinogens, and by-products of lipid peroxidation. AKRs also catalyze the reduction of steroid double bonds (AKR1D, 5β-reductases), and control voltage-gated K+ channels. Since AKRs are involved in the detoxification of drugs and chemical carcinogens, they are generally classified as phase I xenobiotic-metabolizing enzymes [11,15,44].

AKR1B1 catalyzes the reduction of glucose to sorbitol in the presence of NADPH via the polyol pathway. This is followed by the oxidation of sorbitol to fructose using NAD as a cofactor. Under euglycemic conditions, most of the glucose undergoes phosphorylation to glucose-6-phosphate in a reaction catalyzed by hexokinase and less than 3% of glucose is channelized into the polyol pathway. However, under hyperglycemic conditions, the channeling into the polyol pathway is significantly increased to almost 30% glucose with excessive generation of sorbitol implicated in diabetic complications and non-alcoholic fatty liver disease [25,32]. However, there is growing evidence to suggest that glucose is probably not the main substrate of AR, and the enzyme is more efficient in reducing a wide range of aldehydes and their glutathione conjugates formed from lipid peroxidation [21,45]. Reactive oxygen species (ROS) generated during normal metabolism and in pathological conditions induce lipid peroxidation with consequent formation of reactive carbonyl species (RCS) such as 4-hydroxytrans-2-nonenal (HNE) that can deplete reductants including glutathione resulting in damage to cellular macromolecules such as lipids, proteins, and nucleic acids eventually culminating in tissue injury. Although AKRs together with other oxidoreductases can detoxify the RCS to prevent tissue injury, they can trigger ROS signaling [46]. Furthermore, AR-mediated increased flux of sorbitol via the polyol pathway during hyperglycemia can alter NADPH/NADP as well as reduce glutathione/oxidized glutathione (GSH/GSSG) ratios leading to oxidative stress [21,25].

AKR1B10 functions as a multifunctional NADPH-dependent reductase and catalyzes the reduction of a wide range of carbonyl compounds, aldehydes, and xenobiotics including drugs and carcinogenic chemicals such as polycyclic aromatic hydrocarbons (PAH) [47–51]. AKR1B10 catalyzes the transformation of PAH *trans*-dihydrodiols to highly reactive *O*-quinones, with the generation of ROS [52]. Despite its sequence similarity with AKR1B10, AKR1B15 is unique concerning to substrate specificity. AKR1B15 was found to be more catalytically active against a range of ketone and carbonyl substrates compared to AKR1B10. Interestingly, AKR1B15 exhibited high catalytic efficiency with 9-*cis*-retinaldehyde unlike AKR1B10, which prefers *all-trans*-retinaldehyde as a substrate [53].

Benzo[α]pyrene, a PAH, undergoes metabolic activation by the phase I enzymes cytochrome P450 (CYP)1A1/B1 to the highly carcinogenic benzo[α]pyrene-7,8-dihydrodiol, which is oxidized by AKR1A1 to form the corresponding ketol followed by rearrangement and oxidation to *O*-quinone with concomitant generation of ROS [47,54]. Jiang et al. [55] provided experimental evidence to demonstrate the competing roles of CYPs and AKRs in the metabolic activation of (+/−)-7,8-dihydroxy-7,8-dihydrobenzo[a]pyrene (BP-7,8-diol) in human bronchoalveolar H358 cell extracts. While CYP1A1/1B1 induction resulted in the formation of (+/−)-*trans*-7,8-dihydroxy-9alpha,10alpha-epoxy-7,8,9,10-tetrahydrobenzo[a]pyrene (anti-BPDE), AKR1A1 overexpressing cells produced benzo [a] pyrene-7,8-dione (BP-7,8-dione). Co-expression of CYP1A1/1B1 and AKR1A1 generated both the products. Furthermore, when NADPH concentration was increased, anti-BPDE was formed by the action of CYP1A1/1B1, whereas under conditions of higher NAD concentration, The system with a relatively higher concentration of NADPH favored the formation of anti-BPDE via P450 1A1/P450 1B1, AKR1A1 mediated formation of BP-7,8-dione was favored. However, equal amounts of both products were formed under conditions of normal cellular redox state. These findings indicate that metabolic activation of BP-7,8-diol depends on the cellular redox status.

Human AKRs regulate the expression of several genes via *cis-*elements by activating the various response elements in the promoter regions including the osmotic response element (ORE), phorbol ester response (AP-1) element, and the antioxidant response element (ARE) [56–58]. The AKRs respond to different types of stress such as electrophilic stress, oxidative stress, osmotic stress, and steroid hormone stress, and are therefore regarded as stress response genes [59]. AKR1C enzymes are recognized to regulate the expression of several genes via the steroid hormone receptors. AKR1C1 (20α-hydroxysteroid dehydrogenase) reduces progesterone to inactive 20α-hydroxyprogesterone and other progesterones in the endometrium thereby limiting the binding of progestin to the progesterone receptor [60]. AKR1C2 (type 3 3α-HSD/bile acid binding protein) reduces the potent androgen, 5α-dihydrotestosterone (5α-DHT) to the weak androgen 3α-androstanediol and prevents the binding of androgen to the androgen receptor. AKR1C3 catalyzes the activation of androgen Δ4-androstene-3,17-dione, a weak androgen to testosterone, which is then converted to dihydrotestosterone, with resultant enhanced androgen receptor activity in the prostate. AKR1C3 also catalyzes the activation of estrone to 17β-estradiol, and the inactivation of progesterone to 20α-hydroxyprogesterone in the breast with consequent transactivation of the estrogen receptor [61,62].

## AKR Expression in Cancer

Aberrant expression of AKRs has been reported in a broad range of malignancies (Table S1). These include cancers of the adrenal cortex [63], breast [64], cervix [65], colorectum [66–68], liver [69], lung [27,70,71], oral cavity [28], stomach [29], pancreas [72] and glioma [73] to name a few. These studies lend credence to the proposition that AKRs are promising biomarkers for tumor diagnosis and prognosis [26,43,69].

The AKR1B and AKR1C family of proteins have been more strongly associated with cancer development and worse outcomes relative to the other isoforms [11]. Analysis of the Oncomine cancer gene expression database revealed wide variations in the expression of AKR1B1 and AKR1B10 in different cancer types [74]. Overexpression of AKR1B10 was seen in cancers of the lungs and liver associated with a less aggressive clinical course, whereas AKR1B1 was more widely overexpressed in several cancers and associated with shortened patient survival. Overexpression of AKRB10 has been documented in several cancer cell lines including pancreatic adenocarcinoma, medulloblastoma D341, and colon cancer HT29. High expression of AKR1B1 was observed in bladder and renal carcinomas with decreased expression in invasive breast cancer, colorectal cancer (CRC), adrenal cortex, and prostate cancer. On the other hand, high protein expression of AKR1B1 was observed in basal-like breast cancer [30], ovarian, pancreatic, and cervical cancer [75] which may be attributed to post-transcriptional regulation or differences in the clinical status of the patients evaluated. Similarly, overexpression of AKR1B10 was documented in hepatocellular carcinoma [76], breast [31,64,77], pancreatic [78], and lung cancer [79] with significant downregulation in CRC [80].

There are very few reports on the involvement of AKR1B15 in cancer [16,81]. Yuan et al. [82] developed a novel metabolism-related gene signature with AKR1B15 as one of the six genes with the potential to predict overall survival, prognosis, and treatment efficiency for HCC. More research on the role of AKR1B15 in different types of cancer is warranted.

### Liver cancer

Overexpression of AKR1B10 was first observed in hepatocellular carcinoma (HCC) and subsequently reported in a wide range of malignant cell lines and tumor tissues. Increased AKR1B10 expression has been documented in serum and tissue samples of HCC patients [83–85]. High expression of AKR1B10 during the early stages of HCC with subsequent decrease as metastasis supervenes has been documented [86]. Interestingly, overexpression of AKR1B10 in HCC tissues was associated with a favorable prognosis, longer recurrence-free survival (RFS), and disease-specific survival (DSS) [84]. A meta-analysis of 11 studies that included 2747 HCC patients and 2053 controls revealed the high sensitivity and specificity of AKR1B10 as a diagnostic marker for HCC. Notably, unlike AFP, AKR1B10 could predict overall survival and distinguish HCC from benign liver diseases [87]. A large-scale multicenter study involving 273 naïve HCC patients indicated high serum AKR1B10 as an unfavorable prognostic marker that was more sensitive than AFP both independently as well as in combination with TNM stage [69]. While AKR1B10 expression was significantly higher in HCC tumor tissues relative to normal tissues, AKR1B1 expression was not remarkably different between tumor and normal tissues.

### Lung cancer

Increased expression of AKRs has been reported in tobacco-induced cancers. AKRs are known to activate PAH *trans*-dihydrodiol proximate carcinogens from tobacco smoke to toxic and highly reactive *O*-quinones leading to mutational inactivation of p53 [47–50,88]. Increased expression of both AKR1B1 and AKR1B10 was observed in smokers compared to non-smokers. Upregulation of these AKRs has been linked to lung cancer development and progression [89]. Fukumoto et al. [90] demonstrated overexpression of AKR1B10 in smoking-induced non-small cell lung cancer (NSCLC), Kang et al. [91] reported significantly higher expression of AKR1B10 in tumor samples from NSCLC patients compared to patients with benign lung disease and normal controls. They also found a positive correlation between high AKR1B10 levels and smoking history in NSCLC patients. AKR1B10 expression was also higher in the airways of smokers relative to non-smokers as well as in specimens from NSCLC of non-smokers [92]. Simultaneous expression of AKR1B1 was noticed in individual mixed-lineage tumor cells comprised of different histological subtypes of NSCLC underscoring the value of this protein as a candidate target for therapeutic intervention of these tumors [27]. Using a multi-organ microfluidic bionic chip platform, Liu et al. [93] convincingly showed a significant upregulation of AKR1B10 in bone metastasis of NSCLC verifying its efficacy as a valuable serum biomarker of bone metastasis.

### Hormone-dependent tumors

Dysregulation of AKRs expression has been documented in hormone-dependent tumors including prostate cancer and breast cancer. AKR1 and AKR7 have been implicated in the development of prostate cancer [94]. AKR1C3 which plays a key role in peripheral androgen biosynthesis was found to be increased by androgen deprivation in castrate-resistant prostate cancer [95–97]. Furthermore, AKR1C3 which functions as a prostaglandin F (PGF) synthase as well as its isoforms PGF2α and 11β-PGF2α are reported to induce prostate cancer development by preventing the activation of peroxisome proliferator-activated receptor gamma (PPARγ) [98].

Overexpression of AKR1C3 is believed to be involved in hormone-dependent breast cancer by catalyzing the conversion of 4-androstene-3,17-dione to testosterone, which functions as a substrate for the aromatase-mediated synthesis of 17β-estradiol [99,100]. In estrogen-dependent malignancies, AKR1C1 and AKR1C3 catalyze the conversion of progesterone to 20α-hydroxyprogesterone thereby increasing the 17β-estradiol: progesterone ratio [60,101]. Li et al. [102] observed a strong correlation between AKR1B10 and HER2 positivity in ductal carcinoma *in situ* (DCIS), indicating the value of AKR1B10 as a potential diagnostic and prognostic marker for early breast cancers. AKR1B10 is also overexpressed in infiltrating and recurrent breast tumors. The expression of AKR1B10 correlated positively with breast tumor size and nodal metastasis and inversely with disease-free survival (DFS). A positive correlation was seen between high levels of AKR1B10 in serum and tumor tissues in breast cancer patients. Tissue microarray analysis revealed overexpression of AKR1B10 in 84% of breast tumors. Further, AKR1B10 expression was significantly increased in postmenopausal breast tumors, while AKR1B1 was not altered [31]. Serum AKR1B10 is regarded as a novel prognostic marker for breast cancer. AKR1B10 is also overexpressed in cervical, uterine, and endometrial cancers. Furthermore, AKR1B10 expression is associated with keratinization and tumor recurrence in cervical cancer [103].

### Gastric cancer

In gastric cancer, AKR1B1 expression was significantly high and associated with TNM staging indicating its prognostic significance [104]. AKR1B1 expression correlated positively with age, invasion, metastasis, staging, and prognosis of gastric cancer [105]. Elevated expression of AKR1B1 was associated with metastasis and lower overall survival in gastric cancer patients [106]. AKR1B1 was identified as a core gene for the regulation of the immune microenvironment, a biomarker for prognosis, and a potential target for gastric cancer treatment based on weighted gene co-expression network analysis, and data from The Cancer Genome Atlas-stomach adenocarcinoma and GSE62254 [107].

In contrast to AKR1B1, AKR1B10 expression was significantly decreased in gastric cancer and associated with tumor size, stage, and metastasis, suggesting that AKR1B10 may be a reliable prognostic indicator [108]. Using bioinformatic methods, Zhou et al. [29] observed differential gene expression and gene methylation of AKRs in gastric cancer patients based on data from various databases. While the expression levels of AKR1B10, AKR1C1, AKR1C2, and AKR7A3 were significantly low in gastric cancer tissues, AKR6A5 expression was higher relative to normal tissues. AKRB1 expression as well as methylation at cg13801416 was of prognostic value. Using bioinformatics analysis, real-time quantitative polymerase chain reaction (qPCR), and co-immunoprecipitation, assays, Yao et al. [108] convincingly demonstrated that AKR1B10 functions as a tumor suppressor in gastric cancer by inhibiting integrin subunit alpha 5.

### Colorectal cancer

Taskoparan et al. [80] demonstrated that the expression levels of AKR1B1 and AKR1B10 are divergent in colorectal cancer (CRC). AKR1B1 expression was unaltered in primary CRC and increased with disease progression. AKR1B1 has also been implicated in the progression of colitis to colon cancer [109]. In cell lines as well as in mouse models of CRC, the expression of AKR1B1 is believed to differ based on the stage and invasiveness of the tumors. For instance, AKR1B1 expression was higher in invasive tumor cells with Trp53 deletion in CRC mice models compared to non-invasive models [69]. Likewise, AKR1B1 expression was higher in the metastatic SW620 CRC cell line compared to the non-metastatic SW480 and HT29 cells [110]. Low AKR1B1 associated with high AKR1B10 expression was indicative of a good prognosis for CRC. Results from four different CRC datasets revealed that decreased AKR1B10 expression is a good predictor of poor overall survival and DFS [111]. Kropotova et al. [67] reported a significant decrease in AKR1B1 mRNA levels in 88 percent of colorectal tumor samples analyzed. qRT-PCR analysis of metastatic lymph nodes from CRC patients revealed a significantly higher expression of AKR1C3 especially in Dukes’ stage C compared to controls indicating that expression of AKR1C3 is a reliable marker for lymph node metastasis [68].

### Oral and esophageal cancer

Overexpression of AKR1B10 is recognized as an independent biomarker of poor prognosis in oral squamous cell carcinoma (OSCC) [112]. Ko et al. [113] reported high salivary AKR1B10 levels in OSCC patients that correlated with areca quid chewing habit, tumor size, and advanced clinical stage of the disease. Overexpression of AKR1B10 was associated with tumor size, invasion, recurrence, and poor overall survival as well as DFS [28]. In addition to AKR1B10, AKR1C1 and AKR1C3 were also overexpressed in oral cancer cell lines [114]. Overexpression of AKR1B10 and AKR1C2 has been reported in Barrett’s esophagus with further increase with progression to esophageal cancer [115].

### Hematological malignancies

Analysis of the Oncomine database revealed significantly elevated expression of AKR1B1 in leukemias, lymphomas, and melanomas. Overexpression of AKR1B1 in leukemias was associated with TCF3-PBX1 gene fusion and rearrangements of the MLL gene located on chromosome 11q23. A significant overexpression of AKR1B10 was also seen in leukemias [74].

### Other cancers

Differential expression of AKR1B1 was seen in benign and malignant tumors of the adrenal cortex [63]. AKR1B1 expression was significantly low in adrenocortical carcinomas compared to adenomas. In human glioma cells, high expression of AKR1B1 was found to be associated with the upregulation of NORAD (non-coding RNA activated by DNA damage) [116]. The expression of AKR1B10 was 20-fold higher in the cyclophosphamide-resistant D341 MED (4-HCR) medulloblastoma cell line compared to the parental cell line [117]. AKR1B10 was found to be upregulated in pancreatic adenocarcinomas and pancreatic intraepithelial neoplasia [78]. A positive correlation was seen between the high expression of AKR1C3 and the risk of bladder cancer progression and mortality [118].

## AKRs and Cancer Hallmarks

**Figure 2:**
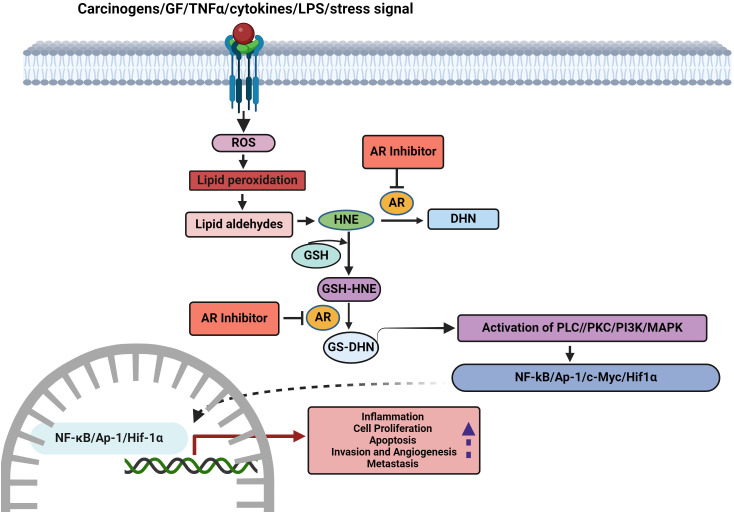
Role of AKRs in the acquisition of hallmarks and scope for therapeutic targeting (LPS-lipopolysaccharide, ROS-reactive oxygen species, HNE-4-hydroxytrans-2-nonenal, GSH-reduced glutathione, PLC-phosphatidylcholine by phospholipase C, PKC-protein kinase C, PI3K-phosphoinositide 3-kinase, MAPK-mitogen-activated protein kinases, NF-κB-nuclear factor-κB, AP1-activator protein-1, HIF1α-hypoxia-inducible factor-1α).

Tumor development is a multistep process that involves the acquisition of eight biological capabilities, referred to as *cancer hallmarks*, namely sustained proliferation, circumventing growth suppressors, cell death evasion, replicative immortality, stimulating angiogenesis, invasion, metastasis, reprogramming cellular metabolism, and overcoming immune destruction. Currently, phenotypic plasticity, epigenetic programming, polymorphic microbiomes, senescent cells, genome instability and mutation, and tumor-promoting inflammation have been incorporated as emerging hallmarks and ‘*enabling characteristics*’ that facilitate the acquisition of cancer hallmarks. [3]. AR ablation by siRNA or inhibition by pharmacological agents has unraveled the oncogenic functions and involvement of the enzyme in the acquisition of several hallmark capabilities of cancer (Fig. 2 and Table S2).

### Effects on cell proliferation

Tumor development is characterized by dysregulated cell cycle control leading to sustained proliferation [119]. Silencing of AKR1B10 inhibited cell growth rate in BT-20 human breast cancer cells and suppressed tumor development in female nude mice [77]. AR inhibition prevented growth factor-induced proliferation of colon cancer cells by arresting cells in the G1 phase, blocking phosphorylation of retinoblastoma protein and cyclin-dependent kinase (cdk)-2, and downregulating the expression of proliferating cell nuclear antigen (PCNA), cyclin D1, cyclin E, cdk4, and c-myc [120,121]. AR inhibition prevented the proliferation of human umbilical vein endothelial cells (HUVECs), as reflected by decreased expression of the proliferation marker, Ki-67 [122]. Both AKR1B1 and AKR1B10 display oncogenic ability. AKR1B1 was shown to induce tumor development by stimulating cell cycle progression in CRC. shRNA knockdown of AKR1B10 in Hep3B HCC cells arrested the cell cycle indicating the role of the enzyme in promoting cell proliferation [123]. Jin et al. [76] provided evidence to show that AKR1B10 facilitates the proliferation of liver cancer cells via phospholipid sphingosine-1-phosphate.

### Effects on apoptosis and autophagy

In HCC cells, shRNA knockdown of AKR1B10 induced apoptosis and suppressed proliferation as evidenced by decreased colony formation, and enhanced chemosensitivity to doxorubicin chemotherapy [124]. The AR inhibitor fidarestat potentiated tumor necrosis factor (TNF)-related apoptosis-inducing ligand (TRAIL) is known to kill tumor cells selectively by inducing the death receptors (DR)-4 and DR-5. This was associated with the downregulation of Bcl-2, Bcl-xL, survivin, XIAP, and FLIP, increased expression of Bax, mitochondrial outer membrane permeabilization (MOMP), resulting in the release of cytochrome c, activation of caspase-3, and cleavage of poly (ADP-ribose) polymerase (PARP). Knockdown experiments revealed that these effects are mediated through activation of the AKT/FOXO3a pathway [125]. In pancreatic adenocarcinoma cell lines, siRNA-knockdown of AKR1B10 induced apoptotic cell death associated with decreased expression of the membrane-bound prenylated protein, KRAS [78].

Autophagy, a cytoprotective mechanism that eliminates misfolded proteins and damaged organelles is intricately linked to apoptosis. In colon cancer, autophagic cell death is recognized to facilitate malignant transformation by enabling cellular nutrient supply, preventing apoptosis, and stimulating drug resistance [126]. Li et al. [127] provided evidence to show that the membrane receptor neuropilin-1 promotes autophagy in colon cancer cells by downregulating AKR1B10 expression indicating that AKR1B10 negatively regulates autophagy. AKR1B10 was demonstrated to repress autophagy in glucose-deprived colon cancer cells by catalyzing the reduction of glyceraldehyde 3-phosphate dehydrogenase (GAPDH) thereby preventing its nuclear import as well as via inhibition of AMPK phosphorylation.

### Effects on angiogenesis, invasion, and metastasis

Intratumoral hypoxia, which promotes angiogenesis and invasion is a driving force for tumor progression [128]. Pharmacological or siRNA-mediated inhibition of AR was demonstrated to inhibit the proliferation of colon cancer cells associated with downregulation of hypoxia-inducible factor-1α (HIF-1α) and its downstream target, vascular endothelial growth factor (VEGF), a potent proangiogenic protein. Additionally, AR inhibition also decreased the expression of markers of inflammation (COX-2 and PGE2), and invasion (matrix metalloproteinase (MMP-2), vimentin, urokinase-type plasminogen activator receptor (uPAR), and lysyl oxidase 2) [129]. In HUVECs, AR inhibition or siRNA ablation blocked invasion and migration induced by VEGF and fibroblast growth factor (FGF). This was associated with decreased expression of interleukin-6 (IL-6), MMP-2, MMP-9, vascular cell adhesion molecule-1 (VCAM-1), and intercellular adhesion molecule-1 (ICAM-1). In the Matrigel plug model of angiogenesis in rats, AR inhibition suppressed angiogenesis as evidenced by significantly reduced vascular infiltration, invasion, migration, and formation of capillary-like vessels, coupled with downregulation of the blood vessel markers CD31 and vWF [122].

Several studies have demonstrated the involvement of AKRs in epithelial-mesenchymal transition (EMT), cell migration, and metastasis. A positive correlation was seen in the expression of AKR1B1 and the mesenchymal transcription factor Twist2. AKR1B1 was found to be a direct transcriptional target of Twist2 in basal-like breast cancer [30]. Tammali et al. [130] provided evidence to show that AR inhibition prevents metastasis of colon cancer by blocking cell adhesion, migration, and invasion. Jin et al. [94] provided compelling evidence to show that AKR1B10 promotes the extravasation of lung cancer cells through the blood-brain barrier leading to brain metastasis. Evaluation of the Genomic Data Commons-The Cancer Genome Atlas (GDC-TCGA) and the International Cancer Genome Consortium (ICGC) databases revealed a significant elevation in AKR1B10 expression in HCC. The increase in AKR1B10 was associated with cell proliferation, migration, invasion, and EMT that contribute to metastasis as evidenced by increased expression of CCND1, E-cadherin, N-cadherin, vimentin, and Twist1 via activation of the PI3K/AKT signaling pathway [131]. Cheng et al. [132] reported elevated expression of AKR1B1 in gastric cancer cells associated with enhanced cell proliferation, migration, and invasion. The transcription factor EBF1 was found to negatively regulate AKR1B1 expression. Knockdown of the transcription factor zinc finger protein521 (ZNF521) induced EBF1 which in turn downregulated AKR1B1 leading to suppression of gastric cancer growth and invasiveness. AKR1B1 overexpression rescued the effects of ZNF521. These findings reveal the involvement of the ZNF521/EBF1/AKR1B1 axis in gastric cancer progression.

## AKRs and Oncogenic ROS Signalling

**Figure 3:**
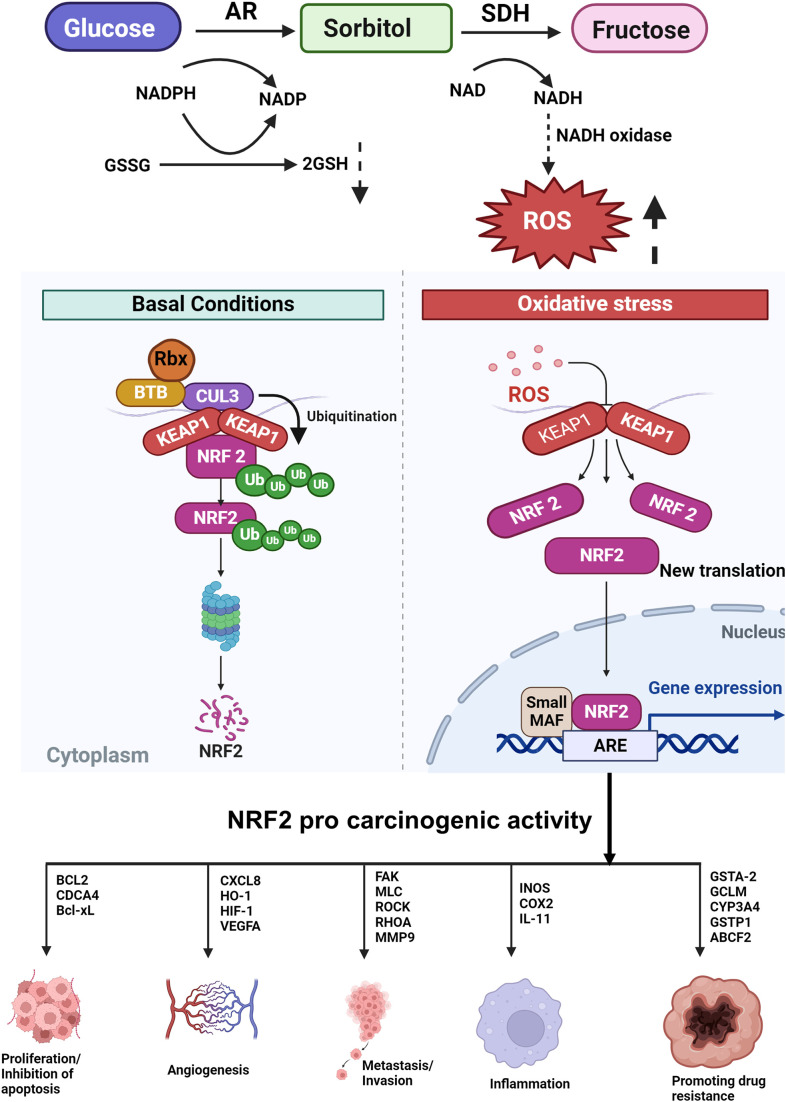
Under normal conditions, there is a basal level of NRF2 expression, while its expression is transiently increased upon exposure to toxic chemicals and ROS as a part of detoxification. However, sustained NRF2 activation confers resistance on cancer cells to anticancer drugs and radiation (SDH-sorbitol dehydrogenase, NADP-nicotinamide-adenine dinucleotide phosphate, NAD-nicotinamide-adenine dinucleotide, ROS-reactive oxygen species, Keap1-Kelch-like ECH-associated protein 1, NRF2-nuclear factor erythroid 2 p45-related factor 2, ARE-antioxidant response element).

There is substantial evidence to indicate that AKR-mediated ROS metabolism activates oncogenic signaling pathways that enable the acquisition of cancer hallmarks [133,134]. Cytokines, growth factors, and lipopolysaccharides trigger ROS-induced lipid peroxidation resulting in the formation of lipid-derived aldehydes such as 4-hydroxynonenal (4-HNE). 4-HNE conjugates with reduced glutathione (GSH) to form 3-glutathionyl-4-hydroxynonenal (GS-HNE). Both HNE and GS-HNE are good substrates for AR. AKRs reduce GS-HNE to the highly reactive GS-4-dihydroxynonane (GS-DHN) that is recognized to activate oncogenic signaling to induce tumorigenesis. AKR1B1 together with GS-DHN is believed to stimulate the phospholipase C/protein kinase C (PLC-PKC) pathway and consequently activate NF-κB. AKR1B1 is also involved in the synthesis of prostaglandins by catalyzing the conversion of PGH2 to prostaglandin F2alpha (PGF2α). Overexpression of AKR1B1 can lead to excessive generation of PGF2α and COX2 resulting in inflammation and tumor development [135,136]. Inhibition of AR prevented growth factor and cytokine-induced cancer cell proliferation, prostaglandin synthesis, COX-2 activity, and activation of NF-κB and PKC. AKR inhibition suppressed ROS generation in cancer cells emphasizing the role of AKR in stimulating ROS-induced oncogenic signaling [25] (Fig. 3). AKR1B1 has been suggested to regulate inflammatory responses and tumorigenesis via activation of ROS, NF-κB, and PGE2 synthesis in CRC [26].

Dysregulation of the phosphoinositide 3-kinase/protein kinase B/mammalian target of rapamycin (PI3K/AKT/mTOR) signaling pathway, a consistent feature of malignant tumors, is intricately linked to ROS generation [137]. Overexpression of AR was found to promote liver inflammation and HCC development via activation of AKT/mTOR signaling by interaction with the kinase domain of AKT1 [138]. In gastric cancer, AKR1B1 overexpression was associated with activation of the AKT-mTOR pathway [83]. AR has been reported to mediate angiogenesis through ROS/PI3K/AKT/AP-1/NF-κB signaling axis [23,122].

AR expression is tightly regulated by the ROS-sensitive transcription factor nuclear factor erythroid 2 p45-related factor 2 (NRF2). The AR promoter region functions as a binding site for the consensus sequence of NRF2. NRF2 regulates the expression of AKR genes. Under normal conditions, NRF2 is sequestered by Kelch-like ECH-associated protein 1 (Keap1), a component of E3 ubiquitin ligase, which causes ubiquitination and proteasomal degradation of NRF2. Under conditions of electrophilic or oxidative stress, NRF2 is detached from Keap1, translocates to the nucleus where it binds to ARE, and transactivates genes that detoxify the stress signals such as AKR1C [45]. NRF2 is reported to regulate AKR1B10 gene regulation. The AKR genes are among the most overexpressed in response to NRF2/Keap-1 signaling. Despite their protective roles against oxidative stress, overexpression of AKR genes constitutes the “*smoking gene*” battery as they can result in the activation of PAHs to carcinogenic metabolites (Fig. 3). Additionally, the upregulation of AKRs via the NRF2 pathway can induce drug resistance [139]. McCleod et al. [140] investigated the link between the expression of AKR and NRF2 activity in 23 cell lines. They found high basal levels of AKRs in cell lines that harbored mutations in the NRF2 pathway indicating that AKRs are biomarkers of NRF2 activity. The sedative propofol reduced the proliferation of BGC823 and GES-1 gastric cancer cells by significantly decreasing the expression of both AKR1B1 and AKR1B10 via the NRF2-mediated polyol pathway.

## AKR and Noncoding RNA

The crosstalk between ROS and the noncoding RNA (ncRNA) is recognized to activate oncogenic signaling and tumor development and progression [141]. Dysregulated microRNA expression has been linked to AKR overexpression. Inhibition of AR was found to prevent the growth of HT29, SW480, and Caco-2 colon cancer cells and nude mice xenografts by downregulating miR-21 expression with concomitant upregulation of programmed cell death 4 (PDCD4), a target of miR-21 by modulation of the ROS/AMPK/mTOR/AP1/4E-BP1 pathway [142]. In addition to PDCD4, AR inhibition also upregulated phosphatase and tensin homolog (PTEN), another miR-21 target, as well as forkhead box O3A (FOXO3a) in HT29, SW480, and Caco-2 colon cancer cells. Silencing of FOXO3a resulted in increasing the expression of AP-1 and miR-21, suggesting that FOXO3a mediates miR-21 repression by inactivating AP-1. Furthermore, AR inhibition prevented growth factor-induced phosphorylation of PI3K/Akt, c-Jun, c-Fos, PTEN, and FOXO3a, as well as DNA-binding activity of AP-1. In nude mice xenografted with human colon adenocarcinoma, AR inhibitor treatment decreased miR-21 expression with a simultaneous increase in the expression of PTEN and FOXO3a [143]. Using integrative bioinformatics mining analysis of NCBI GEO and TCGA databases, Wang et al. [123] identified miR-383-5p as a negative regulator of AKR1B10 in HCC. Dual-luciferase reporter assay provided evidence that miR-383-5p targets the 3’-UTR of AKR1B10 in the post-transcriptional stage leading to its downregulation.

High expression of AKR1B1 and NORAD was seen in glioma cells [116]. Silencing of NORAD prevented proliferation, migration, and invasion, and stimulated apoptosis. On the other hand, co-transfection with AKR1B1 pcDNA3.1 reversed the changes induced by NORAD silencing. In HCC cells, Man et al. [144] demonstrated that downregulation of the epidermal growth factor receptor substrate 15 (EPS15) by the lncRNA EPS15-antisense1 (EPS15-AS1) reduced AKR1B1 expression with consequent stimulation of ferroptosis, inhibition of invasion, and disruption of the redox balance.

## AKR and Drug Resistance

Accumulating evidence has implicated AKRs in resistance to cancer chemotherapeutic agents and anti-hormonal drugs [15,145,146]. AKRs are linked to resistance to several major classes of chemotherapeutic agents including anthracyclines, cisplatin, cyclophosphamide, methotrexate, mitomycin, and vinca alkaloids. Epalrestat is a potent ARI-induced chemosensitivity to doxorubicin and sorafenib through inhibition of AKR1B1 and/or AKR1B10 and blockade of the EMT [145]. Upregulation of AKR1B10 in chemotherapy-resistant medulloblastoma D341 and colon cancer HT29 cancer cell lines underscore the utility of this enzyme as a biomarker of drug resistance. AKRs play a central role in the biosynthesis of androgens and estrogens, as well as in hormone-dependent malignancies. They are also upregulated by hormone ablative therapies that provide a basis for drug resistance. AKR1C3 is implicated in resistance to drugs used for castration-resistant prostate cancer therapy such as androgen receptor signaling inhibitors (ARSI) [146,147].

The involvement of AKR in drug resistance may be a consequence of AKR-catalysed metabolism of chemotherapeutic drugs/steroid hormones or because of the need to eliminate drug-induced cellular stress resulting from the generation of electrophiles or ROS. Additionally, drug-induced cellular stress induces activation of the NRF2/Keap1 pathway leading to feed-forward stimulation of AKRs. Inhibitors of the NRF2/Keap1 pathway and pan-AKR inhibitors may be useful in overcoming drug resistance [45].

Several lines of evidence suggest that AKR inhibitors offer promise in surmounting drug resistance [45]. Various AKR inhibitors have been used for overcoming drug resistance *in vitro*. Although AKR inhibitors potentiate the effect of chemotherapeutic drugs, they are more effective when primed as pretreatment rather than when given concurrently with the chemotherapeutic drug. However, it is important to use pan-AKR inhibitors because inhibition of a single AKR may result in substitution by another AKR. In this context, the non-steroidal anti-inflammatory drugs (NSAIDs) and their analogs have assumed importance in determining the use of AKR inhibitors in surmounting drug resistance. For example, NSAIDs are useful both as isoform-selective inhibitors as well as pan-AKR1C inhibitors. Salicylate is an AKR1C1 selective inhibitor, whereas flufenamic acid, a pan-AKR1C inhibitor can inhibit all four AKR1C isoforms. [148–150]. Recently, Ramana et al. [121] provided evidence to show that AKR1C3 inhibitors restored chemosensitivity in gemcitabine and cisplatin-resistant bladder cancer cells by suppressing androgen signaling.

## AKR Inhibition

Accumulating evidence indicates that inhibition of AR is a pragmatic therapeutic option for cancer both alone and in combination with chemotherapeutic drugs. Since AR has a potential role in mediating inflammation, AKR inhibitors are increasingly used to treat inflammatory diseases such as cancer [35,151]. Repurposing of epalrestat (EPA), a potent AKR1B1/AKR1B10 inhibitor appears promising especially for chemoresistant solid tumors [35,145,152]. Novel methods such as computer modeling and molecular dynamics are being used to design and develop potent ARIs. A comparative analysis of high-resolution crystallographic structures of AKR1B1 and AKR1B10 with reversible inhibitors was undertaken. The active site in both enzymes contained an anion-binding pocket. However, two main differences were observed between AKR1B10 and AR concerning to the involvement of external loops in inhibitor binding. The first difference is the alternative conformation of Trp112 (Trp111 in AR). Secondly, loop A in AKR1B10 was relatively large compared to that in AR with a more loosely packed subpocket [153]. In contrast to AKR1B10, AKR1B15 displayed a more narrower inhibitor selectivity. Among several AKR inhibitors tested against AKR1B15, only JF0064 significantly inhibited AKR1B15 while potent inhibitors of AKR1B1 and AKR1B10 such as tolrestat, epalrestat, sulindac, and sorbinil failed to inhibit AKR1B15. Four Phe residues are believed to contribute to the functional uniqueness of AKR1B15 [53].

**Figure 4:**
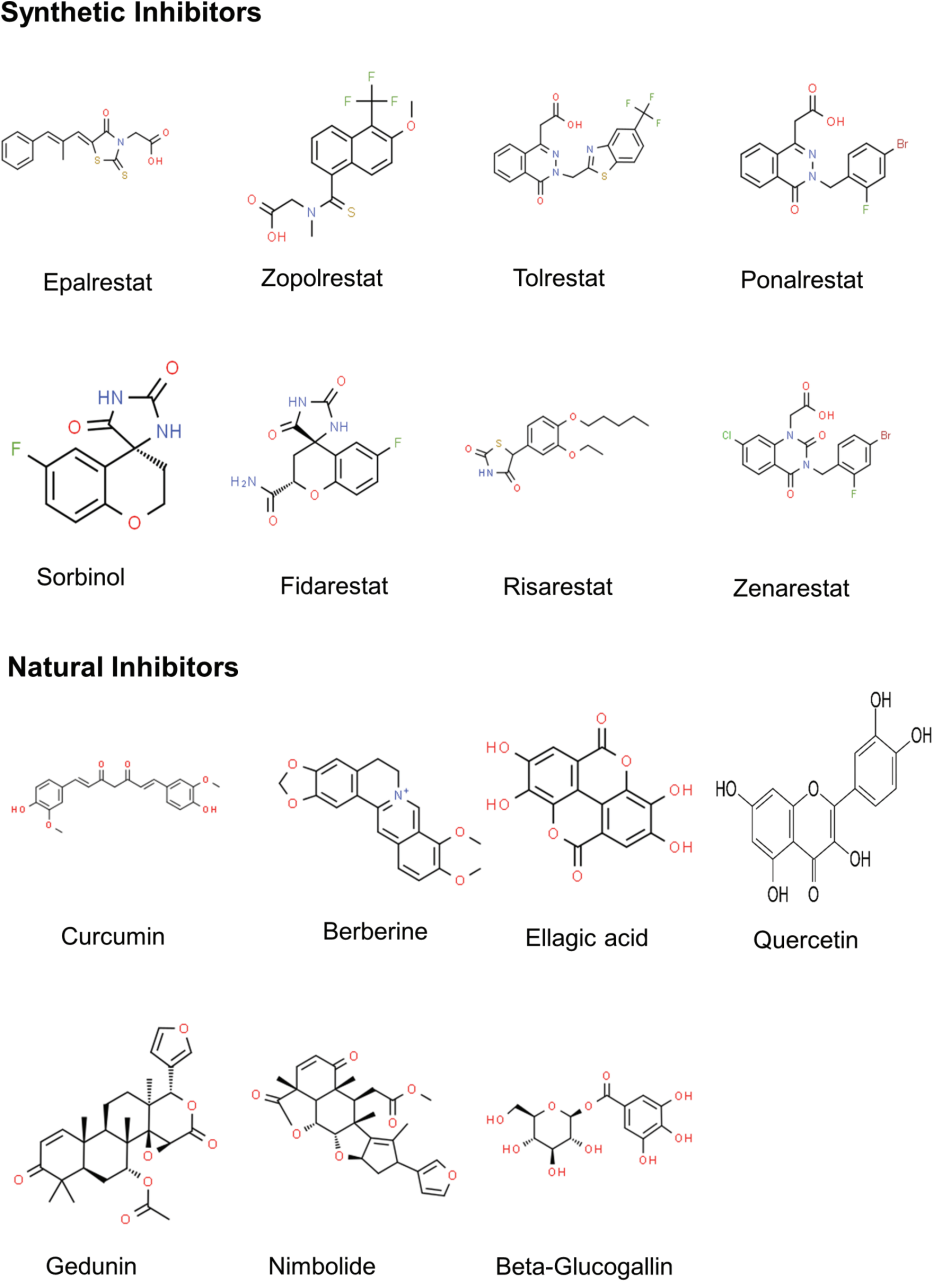
2D structures of major AR inhibitors.

Based on their structure, the AKR inhibitors are classified into three groups, (i) carboxylic acid derivatives such as epalrestat, tolrestat, ponalrestat, zopolrestat, and zenarestat; (ii) spirohydantoins and related cyclic imides such as fidarestat, minalrestat, ranirestat and sorbinil, (iii) phenolic derivatives such as benzopyran-4-one and chalcone [35,154] as well as natural products such as flavonoids, terpenoids, tannins, etc. (Fig. 4 and Table S3). Several ARIs have successfully passed through phase I and II clinical trials but failed in phase III trials due to toxic side effects.

### Epalrestat

Epalrestat (ONO-2235) is the only efficacious and safe ARI approved for the clinical management of diabetic neuropathy. Although epalrestat is not approved by the US Food and Drug Administration for reasons unknown, it is marketed and used in Japan, India, and China. Despite not being extensively used in frontline therapy of diabetic neuropathy, epalrestat continues to be a mainstay as a disease-modifying agent [155].

Epalrestat was shown to inhibit the homologous enzymes AKR1B1 and AKR1B10 [145,156]. The compound has an essential 2-thioxo-4-thiazolidinone motif and regulates intracellular carbonyl species. Epalrestat is orally active and capable of penetrating the blood-brain barrier. There is ample experimental evidence for the anticancer effects of epalrestat in a wide range of malignancies alone and combination with chemotherapeutic agents. Epalrestat enhances the chemosensitivity of cancer cells to doxorubicin by inhibiting the activity of AKR1B1 and/or AKR1B10 as well as by preventing epithelial-mesenchymal transition [145]. Epalrestat has been reported to suppress the proliferation and migration of HeLa cervical cancer cells by inhibiting AKR1B1 [65]. Epalrestat restored the chemosensitivity of lung cancer cells resistant to epidermal growth factor receptor (EGFR), and tyrosine kinase inhibitor (TKI), in addition to delaying resistance in a murine lung cancer xenograft model [157]. A two-pronged minimalist nano platform that combined photodynamic therapy (PDT)-mediated tumor killing and epalrestat-mediated EMT blockade was demonstrated to effectively prevent metastasis of basal-like breast cancer [158].

Knockout of AKR1B1 by the CRISPR/Cas9 technology prevented proliferation, migration, and invasion in the HeLa cell line. Interestingly, epalrestat also produced the same effect as AKR1B1 knockout suggesting its therapeutic potential in cervical cancer [65]. However, the short half-life and hydrophobicity of epalrestat are bottlenecks in using it as a standard-of-care cancer treatment. Targeted co-delivery of epalrestat with doxorubicin was able to achieve synergistic chemotherapeutic efficacy and improve half-life and bioavailability. To this end, doxorubicin was co-loaded into redox-sensitive prodrug micelles. The nanoencapsulated formulation when introduced into mammary carcinoma cells induced cell cycle arrest, apoptosis, and significantly downregulated CD44 receptor expression, a major contributing factor of metastasis [159].

### Fidarestat

Fidarestat is another ARI that has shown a high degree of efficacy as a potential anticancer agent. In SW-480, HT29, and HCT116 CRC cells, fidarestat enhanced the expression of Nrf2. Additionally, in SW480 cells, fidarestat increased Nrf2-DNA binding activity as well as the levels of heme oxygenase-1 (HO-1), NADP(H) quinone oxidoreductase 1 (NQO1), superoxide dismutase, and catalase. In EGF-stimulated CRC cells, fidarestat potentiated EGF-induced Nrf2 and upregulated the expression of peroxisome proliferator-activated receptor gamma coactivator (PGC)-1alpha, transcription factor A, mitochondrial (TFAM), and COX-IV and attenuated DNA damage as seen by decreased levels of 8-hydroxy-2'-deoxyguanosine. Likewise, fidarestat treatment significantly increased Nrf2 and HO-1 levels in nude mice bearing xenografted tumor tissues. Further, fidarestat modulated phosphorylations of AMPK and mTOR as well as p53 expression in EGF-administered cells. Taken together, these results suggest that fidarestat modulates the Nrf2/HO-1/AMPK pathway to regulate mitochondrial biogenesis [160].

Fidarestat administered in drinking water significantly reduced azoxymethane (AOM)-induced premalignant lesions in the colon of C57BL/KsJ-db/db obese mice accompanied by downregulation of PKC-β2, AKT, COX-2, and iNOS in the colonic mucosa, and decreased serum levels of IL-1α, IP-10, MIG, TNF-α and VEGF. Further, in high glucose-treated HT29 colon cancer cells, fidarestat downregulated COX-2, iNOS, XIAP, survivin, β-catenin, and NF-κB [161]. Fidarestat was demonstrated to potentiate TRAIL-induced apoptosis by regulating the AKT/PI3K-dependent activation of FOXO3a [125]. Fidarestat induced autophagy in HT-29 and SW-480 CRC cells as reflected by high levels of LC3 II relative to LC3A/BI [162]. Fidarestat prevented angiogenesis by inhibiting endothelial cell differentiation, invasion, migration, expression of pro-angiogenic factors, and inactivation of AKT [122]. In another study, fidarestat was demonstrated to inhibit metastasis of colon cancer to the liver of nude mice by blockade of invasion, migration, adhesion, and angiogenesis [130].

### Sorbinil

The AKR inhibitors sorbinil or zopolrestat blocked growth factor-induced binding activity of the transcription factor E2F1, phosphorylation of pRb, expression of cyclins and CDKs, generation of ROS, and activation of the PI3K/AKT pathway in colon cancer cells [121]. The formation of AOM-induced preneoplastic aberrant crypt foci (ACF) was significantly reduced in BALB/c mice treated with sorbinil as well as in AR-null mice. This was accompanied by the downregulation of nitric oxide synthase, cyclooxygenase-2, cyclin D1, and beta-catenin with reduced phosphorylation of PKCβ2 and NF-κB binding protein implicating the involvement of AR in mediating ACF formation [120]. Sorbinil treatment prevented FGF and platelet-derived growth factor (PDGF)-induced growth of Caco-2 colon cancer cells and attenuated COX-2, PGE2, NF-κB activation, and PKC as well as phosphorylation of PKC-beta2. Further, AR inhibition blocked HNE-or GS-HNE-induced upregulation of COX-2 and PGE [12,163].

### Ponalrestat

The AKR inhibitor, ponalrestat (statil) is reported to inhibit cancer cachexia by suppressing IL-1 expression [164] Ponalrestat was found to suppress the proliferation of breast and lung cancer cells by downregulating the expression of AKR1B10 [165]. Kavitha et al. [166] investigated the anticancer effects of spirohydantoin compounds in leukemic cells. They found that these compounds induce mitochondrial apoptosis associated with DNA fragmentation, upregulation of p53, and BAD, downregulation of BCL2, Ku70, and Ku80, activation of caspase-3 and -9, and cleavage of PARP.

### Other synthetic small molecular inhibitors

A series of novel quinazolinones (1–21) were synthesized and tested for ALR2 inhibitory activity. Compound 15 exhibited high ALR2 inhibition that was 7.7 fold compared to epalrestat [167]. Of the fifteen tetrazole-hydrazone hybrids designed and synthesized, compound 4 (2-[(1-(4-Hydroxyphenyl)-1H-tetrazol-5-yl)thio]-N'-(4-fluorobenzylidene) acetohydrazide) exhibited strongest AR inhibitory activity comparable to epalrestat without cytotoxicity to normal NIH/3T3 cells [168].

Han et al. [169] designed and synthesized derivatives of quinazolin-4(1H)-one for selective inhibition of AKR1B1. Structure-activity studies revealed that the N1-acetic acid and N3-benzyl groups with electron-withdrawing substituents on the quinazolin-4(1H)-one scaffold enhanced the selectivity and efficiency of AKR1B1 inhibitors. A novel series of 3,4-dihydroquinolin-2(1H)-one derivatives were designed as potent and selective dual inhibitors of AKR1B1 and ROS. Of these, the phenolic 3,5-dihydroxyl compound 8b showed the highest antioxidant activity equivalent to that of Trolox [170].

Novel small molecule derivatives of 2,4-thiazolidinedione (2,4-TZD) synthesized by incorporation of the bioactive scaffolds, benzothiazole heterocycle, and nitrophenacyl moiety were found to exhibit potent AR inhibitory activity [171]. Selective inhibition of AKR1B10 was attempted by synthesizing a series of polyhydroxy steroids. Compound 6 displayed selective inhibition for both AKR1B1 and AKR1B10. Structure-activity studies revealed that the C-19 hydroxyl group can significantly enhance selective inhibition of AKR1B10 [172].

HMPC (7-hydroxy-2-(4-methoxyphenylimino)-2H-chromene-3-carboxylic acid benzylamide) is a potent but nonselective competitive inhibitor of AKR1B10. Derivatives of HPMC were synthesized to enhance selectivity and potency. Among the HMPC derivatives synthesized by removal of the 4-methoxyphenylimino moiety and replacement of the benzylamide with phenylpropylamide, compounds 4c and 4e exhibited greater AKR1B10 inhibitory activity and selectivity compared to HMPC. These compounds significantly suppressed the proliferation, migration, and metastasis of A549 lung cancer cells as well as their cisplatin-resistant counterparts [70].

A series of derivatives containing halophenoxyacetic acid moiety and many bromine (Br) atoms on the aryl moiety were synthesized from the potent AR inhibitor, IDD388. The incorporation of Br atoms reduced AKR1B1 inhibitory activity but enhanced AKR1B10 inhibitory potency. MK204 which forms a strong halogen bond with the enzyme exhibited the highest potency and AKR1B10 selectivity [173].

### Endogenous substances

AKRs are selective targets of cyclopentenone prostaglandin A1 (PGA1), an eicosanoid with anti-inflammatory and antiproliferative properties. PGA1 was shown to covalently bind to Cys299 near the active site of AKR, while His111 and Tyr49, are involved in the orientation of PGA1. Modification of AKR1B10 by biotinylated PGA1 resulted in loss of enzyme activity. Further, PGA1 induced the accumulation of doxorubicin, an AKR substrate in lung cancer cells with consequent cell cycle arrest. These findings characterize PGA1 as an AKR inhibitor that can effectively counter cancer chemoresistance [174]. Other endogenous substances that inhibit AKRs, particularly, AKR1B10 include steroid hormones, bile acids, isolithocholic acid, and androst-4-ene-3,6-dione [175,176].

### NSAIDs and other potential drug candidates

Endo et al. [177] demonstrated selective inhibition of AKR1B10 by NSAIDS, N-phenylanthranilic acids, and glycyrrhetic acid. Molecular docking studies revealed that the side chain of Val301 and hydrogen bonds between Val301, Gln114, and Ser304 are crucial for the selective inhibitory potency of the NSAIDs to target AKR1B10 and function as anticancer agents. Cemtirestat (3-mercapto-5H-[1,2,4]-triazino[5,6-b]indole-5-acetic acid), a highly efficient ARI and potent antioxidant was designed and patented. The cytotoxicity of cemtirestat was significantly lower than that of epalrestat. Based on *in silico* predictions, *in vitro* and *in vivo* assays, cemtirestat was found to be a safe drug with therapeutic potential that can progress to clinical trials [178].

*Trans*-(±)-kusunokinin ((±)KU), a synthetic antiproliferative agent that inhibits the growth of several malignant tumor cells both *in vitro* and *in vivo*, was shown to be a selective inhibitor of AKR1B1 [179–181]. (±)KU exerted more potent cytotoxic effects on triple-negative breast and ovarian cancer cells compared to epalrestat [182]. (±)KU was shown to bind to AKR1B1 and inhibit oxidative stress as well as the migration, EMT, and invasion of breast cancer cells.

### AKR inhibitors of plant origin

Although many synthetic AKR inhibitors have been developed, many of these have been withdrawn following clinical trials due to low efficacy and safety concerns. The discovery and development of AKR inhibitors from natural sources have therefore gained the focus of research attention. Veeresham et al. [182] classified AKR inhibitors of plant origin into different groups based on their chemical structure, which include flavonoids, tannins, alkaloids, terpenoids, coumarins, and miscellaneous compounds.

A variety of flavonoids abundantly present in fruits, vegetables, herbs, and spices are known to inhibit AKR. Varma et al. [183] demonstrated that quercetin, quercetrin, and myrcitrin are effective inhibitors of AKR. Rutin is one of the commonly found dietary flavonols with AKR-inhibiting activity [184]. Xanthohumol, isoxanthohumol, and 8-prenylnaringenin flavonones found in hops (*Humulus lupulus*) that exhibit anticancer activity are potent inhibitors of both AKR1B1 and AKR1B10 but do not affect the activity of AKR1A1 [185]. Zemanova et al. [186] examined 40 different phenolics and alkaloids for their potential to inhibit AKR1B10. They identified flavones such as apigenin, luteolin, and 7-hydroxyflavone as potent inhibitors of AKR1B10 based on IC50, selectivity, and mechanism of action. These compounds were found to significantly inhibit AKR1B10-catalysed reduction of the chemotherapeutic agent daunorubicin without causing cytotoxicity.

Among the various tannins investigated, beta-glucogallin, a major component of gooseberry (*Emblica officinalis*) and precursor of ellagic acid in strawberry and raspberry, is a potent AKR inhibitor [187]. Recently, Suyanto et al. [188] provided evidence to show that β-glucogallin prevents the proliferation and migration of cholangiocarcinoma cells. A hydrolyzable tannin pentagalloyl glucose isolated from the bark of Rhus verniciflua displayed potent inhibition of human recombinant AR [189]. Alkaloids are secondary metabolites produced by plants that offer immense scope for the development of various drugs including anticancer agents [190]. The alkaloid dehydrocorydaline isolated from methanolic extract of the tuber of *Corydalis turtschaninovii* that prevents metastasis of NSCLC via suppression of MMPs and BCL2 is also an AKR inhibitor [191,192]. Berberine, another plant alkaloid that exhibits remarkable anticancer properties [193] inhibits AR activity *in vivo* [194].

Terpenoids also known as isoprenoids that exhibit a wide array of pharmacological effects including antiproliferative activity, are potent AKR inhibitors [185,195]. The triterpenoids or limonoids from the neem tree (*Azadirachta indica*) display potent anticancer properties [196]. Two neem limonoids, gedunin and nimbolide have been demonstrated to abrogate oncogenic signaling pathways and prevent the acquisition of cancer hallmarks by inhibiting AKR. Administration of gedunin, a neem limonoid, prevented angiogenesis in hamster oral tumors via the inactivation of AR. The mechanism involved the blockade of PI3K/Akt and NF-κB pathways and the downregulation of the expression of the oncomiR, miR-21, HIF-1α, and VEGF [197]. In SCC131 oral cancer cells, gedunin alone and in combination with epalrestat prevented cancer hallmarks presumably by inhibiting AR-mediated ROS signaling and co-inactivation of Akt, Erk, and IKK/NF-κB signaling, with the combination being more effective than the single agents. Cotreatment with gedunin and epalrestat induced G1/S phase cell cycle arrest, and cell death initially by autophagy and subsequently by apoptosis. Furthermore, the combination inhibited angiogenesis in both SCC131 cells as well as in Eahy926 endothelial cells [198]. Nimbolide, another neem limonoid was demonstrated to inhibit angiogenesis in breast cancer cells as well as in a xenografted nude mouse model of breast cancer by AR inhibition with the consequent abrogation of the insulin-like growth factor-1 (IGF-1)/PI3K/Akt and HIF-1α/VEGF signaling. Further, nimbolide was shown to regulate AR and IGF-1/PI3K/Akt signaling by epigenetic modulation. Most importantly, co-administration of nimbolide with metformin or the chemotherapeutic drugs tamoxifen/cisplatin was found to be more efficacious than single agents in abrogating IGF-1/PI3K/Akt/AR signaling suggesting that AKR inhibitors show promise in chemosensitization [199].

Coumarins that contain a benzopyran-2-one or chromen-2-one ring system, are found in various natural products. Kawanishi et al. [200] listed 41 coumarins that exhibit AR inhibitory activity. Angular dihydropyranocoumarins isolated from the flowers of *Peucedanum japonicum* (Umbelliferae) exhibited significant AKR1C1 inhibitory activity in A549 human non-small-cell lung cancer cells [201]. Nodakenin and scopoletin are naturally occurring coumarins that are reported to exert anticancer effects as well as AKR inhibitory activity [202,203]. A wide variety of dietary compounds identified as AKR inhibitors are also anticancer agents. These include spinach, cinnamon, lemon, basil, fenugreek, and curcumin among several others [154,204].

## Conclusions

Aldo-keto reductases are a superfamily of enzymes that play crucial roles in various cellular processes. AKRs play a significant role in cancer biology, contributing to metabolic reprogramming, drug resistance, and tumorigenesis. Targeting AKRs through various strategies including the use of synthetic or natural ARIs holds promise for the development of novel cancer therapies. Further research is needed to elucidate the specific roles of individual members of the AKR family in different cancer types, identify biomarkers of AKR activity, and optimize therapeutic approaches to effectively target AKRs in cancer treatment.

In general, downregulation of AKRs is less common in malignancies compared to overexpression. While AKR1B1 is significantly elevated in liver and lung cancer, it is more widely overexpressed in a range of malignancies and is associated with worse outcomes. Given the significant association between diabetes and increased cancer risk, AKR1B1 due to its involvement in the polyol pathway and oncogenic signaling could presumably emerge as a vital link in the context of coincident disease. On the other hand, AKR1B10 is implicated in cell proliferation and chemoresistance and is less widely distributed in normal tissues relative to AKR1B1. These characteristics render AKR1B10 as a promising tumor marker and therapeutic target in specific tumors.

Despite convincing evidence implicating the role of AKRs in cancer, more intensive clinical studies are warranted on a broader range of malignant tumors, especially concerning the different isoforms. Clinical research has largely focused on AKR1B1 and AKR1B10 with very little emphasis on other members that could shed light on specific pathways and cellular processes that are specific to cancer type and tissue of origin.

Although many preclinical studies have demonstrated the efficacy of AKR inhibitors as anticancer agents, only a few have entered clinical trials. It is important to identify AKR inhibitors that exhibit high potency, safety, and isoform selectivity. A major bottleneck in the development of selective AKR inhibitors is the structural similarity among AKR family proteins. It is important to unravel subtle structural differences that can prevent cross-inhibition and facilitate selective inhibition. Natural products with their rich repertoire of chemical entities offer promise in the design and development of novel AKR inhibitors.

## Supplementary Materials







## Data Availability

Not applicable.

## Abbreviation

AKR: Aldo-keto reductase
AKT: Protein kinase B
ARE: Antioxidant response element
AR: Aldose reductase
ARI: Aldose reductase inhibitor
COX: Cyclooxygenase
CRC: Colorectal cancer
CYP: Cytochrome P450
EGF: Epidermal growth factor
EGFR: Epidermal growth factor receptor
EMT: Epithelial-mesenchymal transition
FGF: Fibroblast growth factor
GSH: Reduced glutathione
GS-HNE: 3-Glutathionyl-4-hydroxynonenal
GSSG: Oxidized glutathione
HCC: Hepatocellular carcinoma
HIF-1α: Hypoxia-inducible factor-1α
HNE-4: Hydroxytrans-2-nonenal
HO-1: Heme oxygenase-1
IGF-1: Insulin-like growth factor-1
Keap1: Kelch-like ECH-associated protein 1
mTOR: Mammalian target of rapamycin
NAD: Nicotinamide-adenine dinucleotide
NADP: Nicotinamide-adenine dinucleotide phosphate
NF-κB: Nuclear factor-κB
NRF2: Nuclear factor erythroid 2 p45-related factor 2
NSAIDS: Non-steroidal anti-inflammatory drugs
ORE: Osmotic response element
PCNA: Proliferating cell nuclear antigen
PI3K: Phosphoinositide 3-kinase
PKC: Protein kinase C
ROS: Reactive oxygen species
TNF: Tumor necrosis factor
VEGF: Vascular endothelial growth factor

## Supplementary Materials

The supplementary material is available online at https://doi.org/10.32604/or.2024.049918.
