# Supplementary material for: Aldo-keto reductases: Role in cancer development and theranostics
Source: Oncol Res. 2024 Jul 17;32(8):1287–308. doi: 10.32604/or.2024.049918 (PMC11267078; doi:10.32604/or.2024.049918)
Supplement: Supplementary file 1 [file OncolRes-32-49918-s001.docx]

SUPPLEMENTARY TABLE 1. Summary of altered expression of AKR isoforms in different cancers

| **Cancer site** | **Expression of AKR isoforms** | | | | | | | | **Reference(s)** |
| --- | --- | --- | --- | --- | --- | --- | --- | --- | --- |
|  | **AKR1B1** | **AKR1B10** | **AKR1B15** | **AKR1C1** | **AKR1C2** | **AKR1C3** | **AKR6A5** | **AKR7A3** |  |
| Adrenal cortex | Low |  |  |  |  |  |  |  | [63] |
| Bladder | High | High |  |  |  | High |  |  | [74,118] |
| Breast | Low  High in BLBC | High |  |  |  | High |  |  | [30,31,59,99-102] |
| Cervix | High | High |  |  |  |  |  |  | [74,65,103] |
| Colorectum  Lymph node metastasis from CRC | Low | Low |  |  |  | High |  |  | [66,67,74]  [68] |
| Endometrium |  | High |  | High |  | High |  |  | [60,103] |
| Esophagus | High | High |  |  | High |  |  |  | [74,115] |
| Glioma | High | High |  |  |  |  |  |  | [73,74,116,117] |
| Head & Neck | High | Low |  |  |  |  |  |  | [74] |
| Kidney | High |  |  |  |  |  |  |  | [74] |
| Leukemias | High | High |  |  |  |  |  |  | [74] |
| Liver |  | High | High |  |  |  |  |  | [69,74] |
| Lung | High | High |  |  |  |  |  |  | [27,70, 71,89,90] |
| Oral cavity |  | High |  | High |  | High |  |  | [28,113,114] |
| Pancreas |  | High |  |  |  |  |  |  | [72,74] |
| Prostate | Low |  |  |  |  | High |  |  | [74,96,97] |
| Stomach | High | Low |  | Low | Low |  | High | Low | [27,29,104-107] |
