# Supplementary material for: Aldo-keto reductases: Role in cancer development and theranostics
Source: Oncol Res. 2024 Jul 17;32(8):1287–308. doi: 10.32604/or.2024.049918 (PMC11267078; doi:10.32604/or.2024.049918)
Supplement: Supplementary file 2 [file OncolRes-32-49918-s002.docx]

**SUPPLEMENTARY TABLE 2.** Effect of AR inhibition/ablation on cancer hallmarks

| **Cancer Hallmark** | **Cellular/Animal Model** | **Molecules/Processes/Signalling pathways modulated** | **Reference(s)** |
| --- | --- | --- | --- |
| Cell proliferation | HT29, SW480 and HCT-116 colon cancer cells | - G1 phase cell cycle arrest - Reduced pRb phosphorylation downregulation of PCNA, cyclin D1, cyclin E, cdk2, cdk4, and c-myc - Prevented growth factor-induced activation of PI3K/Akt & ROS generation | [121] |
|  | AOM-induced colon cancer in BALB/c mice, AR-null mice | - Downregulation of cyclin D1 & β-catenin | [120] |
|  | HUVECs | - Downregulation of Ki-67 | [122] |
|  | Hep3B HCC cells | - Cell cycle arrest | [123] |
| Apoptosis evasion | HT-29, SW-480 and HCT-116 colon cancer cells | - Potentiation of TRAIL-induced apoptosis - Induction of death receptors (DR)-4 & DR-5. Downregulation of Bcl-2, Bcl-xL, survivin, XIAP, and FLIP - mitochondrial outer membrane permeabilization - Upregulation of Bax, (MOMP) - Release of cytochrome c - Caspase-3 activation - PARP cleavage - Activation of AKT/FOXO3a pathway | [125] |
|  | Panc10.05 Pancreatic adenocarcinoma cells line | - Downregulation of KRAS, ERK, MEK | [78] |
|  | HT29, SW480 and Caco-2 colon cancer cells  Nude mice xenografts | - Downregulation of miR-21 - Upregulation of PDCD4, PTEN, FOXO3a - Modulation of ROS/AMPK/mTOR/AP1/4E-BP1 pathway | [142] |
|  | HT29, SW480, and Caco-2 colon cancer cells  HT29 xenografted nude mice | - Prevented growth factor-induced phosphorylation of PI3K/Akt, c-Jun, c-Fos, PTEN, and FOXO3a, DNA-binding activity of AP-1. - Increased miR-21 expression with simultaneous decrease in the expression of PTEN and FOXO3a | [143] |
| Angiogenesis | HT29, SW480, and Caco-2 colon cancer cells | - Downregulation of HIF-1α & VEGF | [129] |
|  | Rat Matrigel plug model | - Reduced vascular infiltration, invasion, migration, and formation of capillary-like vessels - Downregulation CD31 and vWF | [122] |
| Inflammation | HT29, SW480, and Caco-2 colon cancer cells | - Downregulation of COX-2 & PGE2 | [129] |
|  | HUVECs | - Downregulation of IL-6 | [122] |
|  | AOM-induced colon cancer in BALB/c mice, AR-null mice | - Downregulation of iNOS & COX-2 - Decreased phosphorylation of PKCβ2 and NF-kB p65 | [120] |
| Invasion & metastasis | HT29, SW480, and Caco-2 colon cancer cells | - Downregulation of MMP-2, vimentin, uPAR, lysyl oxidase 2 | [129] |
|  | HUVECs | - Downregulation of MMP-2, MMP-9, V-CAM, ICAM-1 | [122] |
|  | HT29; KM20 colon cancer cells | - Blockade of cell adhesion, migration, invasion | [130] |
