# Supplementary material for: Aldo-keto reductases: Role in cancer development and theranostics
Source: Oncol Res. 2024 Jul 17;32(8):1287–308. doi: 10.32604/or.2024.049918 (PMC11267078; doi:10.32604/or.2024.049918)
Supplement: Supplementary file 3 [file OncolRes-32-49918-s003.docx]

**SUPPLEMENTARY TABLE 3.** Mechanism of anticancer activity of some known and potential AKR inhibitors

| **Inhibitor** | **Isoform selectivity** | **Mechanism of anticancer activity** | **Reference(s)** |
| --- | --- | --- | --- |
| Epalrestat | AKR1B1 & AKR1B10 | - Suppression of proliferation, migration, & invasion in HeLa cervical cancer cells | [65] |
|  |  | - Enhanced chemosensitivity to doxorubicin | [145] |
|  |  | - EMT blockade | [145] |
|  |  | - Restoration of chemosensitivity to EGFR TKI in lung cancer cells | [157] |
|  |  | - Delayed chemoresistance in lung cancer cells and murine xenograft model | [157] |
|  |  | - Prevention of metastasis by EMT blockade in basal-like breast cancer cells | [158] |
|  |  | - Cell cycle arrest, apoptosis induction, CD44 receptor downregulation in mammary carcinoma cells | [159] |
| Fidarestat | AKR1B1 & AKR1B10 | - Enhanced expression of NRF2, HO-1, NQO1, SOD, catalase, PGC-1 alpha, TFAM, COX-IV - Attenuated DNA damage - Modulated phosphorylation of AMPK & mTOR in CRC cells - Modulated Nrf2/HO-1/AMPK pathway in nude mice xenograft model | [160] |
|  |  | - Downregulated PKC-β2, AKT, COX-2 and iNOS in colon cancer cells - Decreased serum IL-1α, IP-10, MIG, TNF-α and VEGF in AOM induced C57BL/KsJ-db/db obese mice - Downregulated COX-2, iNOS, XIAP, survivin, β-catenin and NF-kB in HT29 colon cancer cells | [161] |
|  |  | - Potentiated TRAIL-induced apoptosis by regulating the AKT/PI3K-dependent activation of FOXO3a | [125] |
|  |  | - Induced autophagy in HT-29 and SW-480 CRC cells | [162] |
|  |  | - Prevented angiogenesis by AKT inactivation | [122] |
|  |  | - Inhibited colon cancer metastasis to the liver by blockade of invasion, migration, adhesion, and angiogenesis in mouse models | [130] |
| Sorbinil |  | - Blocked E2F1 binding activity, pRb phosphorylation, expression of cyclins and CDKs, ROS generation, and activation of PI3K/AKT pathway in colon cancer cells | [121] |
|  |  | - Reduced AOM-induced ACF - Downregulated NOS, COX-2, cyclin D1 and β-catenin - Reduced phosphorylation of PKC-β2 and NF-kB binding protein in BALB/c mice and AR-null mice | [120] |
|  |  | - Prevented GF-induced proliferation - Inhibited COX-2, PGE(2), NF-kB activation, PKC-β2 phosphorylation | [163] |
| Ponalrestat |  | - Suppression of IL-1 expression | [145] |
|  |  | - Inhibited proliferation of breast and lung cancer cells | [166] |
| HPMC deriva-tives 4c & 4e | AKR1B10 | - Suppressed proliferation, migration, and metastasis in A549 lung cancer cells | [70] |
| IDD388 derivative with Br incorporated | AKR1B10 | - To be investigated | [173] |
| MK-204 | AKR1B10 | - To be investigated | [173] |
| Cyclopentenone prostaglandin A(1) (PGA(1)) | AKR1B10 | - Cell cycle arrest - Counters chemoresistance in lung cancer cells | [174] |
| NSAIDs | AKR1B10 | - To be investigated | [177] |
| Cemtirestat | AKR1B1 | - To be investigated | [178] |
| *Trans*-(±)-kusunokinin | AKR1B1 | - Prevents proliferation, migration, EMT, and invasion of breast cancer cells in breast and ovarian cancer cells | [179-181] |
| Beta-glucogallin | AKR1B1 | - Prevents proliferation & migration of cholangiocarcinoma cells | [188] |
| Gedunin | AR | - Prevents proliferation, apoptosis evasion, angiogenesis, and invasion, by abrogation of PI3K/Akt, NF-kB, VEGF signaling, and downregulation of miR-21 in oral cancer cells and hamster oral tumors | [197,198] |
| Nimbolide | AR | - Inhibits angiogenesis in breast cancer cells and xenografted nude mouse model by abrogation of IGF-1/PI3K/Akt and VEGF signaling | [199] |
